# A DAG-based comparison of interventional effect underestimation between composite endpoint and multi-state analysis in cardiovascular trials

**DOI:** 10.1186/s12874-017-0366-9

**Published:** 2017-07-04

**Authors:** Antje Jahn-Eimermacher, Katharina Ingel, Stella Preussler, Antoni Bayes-Genis, Harald Binder

**Affiliations:** 1 Institute of Medical Biostatistics, Epidemiology and Informatics, University Medical Center Johannes Gutenberg-University Mainz, Obere Zahlbacher Str. 69, Mainz, 55131 Germany; 2 Heart Failure Clinic, Cardiology Service, CIBERCV, Department of Medicine, UAB, Hospital Universitari Germans Trias i Pujol, Carretera del Canyet, Badalona, Barcelona, 08916 Spain; 3 Institute for Medical Biometry and Statistics, Faculty of Medicine and Medical Center - University of Freiburg, Stefan-Meier-Str. 26, Freiburg, 79104 Germany

**Keywords:** Composite endpoint, Recurrent events, Multi-state models, Hospital admissions, Bias, Cardiovascular

## Abstract

**Background:**

Composite endpoints comprising hospital admissions and death are the primary outcome in many cardiovascular clinical trials. For statistical analysis, a Cox proportional hazards model for the time to first event is commonly applied. There is an ongoing debate on whether multiple episodes per individual should be incorporated into the primary analysis. While the advantages in terms of power are readily apparent, potential biases have been mostly overlooked so far.

**Methods:**

Motivated by a randomized controlled clinical trial in heart failure patients, we use directed acyclic graphs (DAG) to investigate potential sources of bias in treatment effect estimates, depending on whether only the first or multiple episodes are considered. The biases first are explained in simplified examples and then more thoroughly investigated in simulation studies that mimic realistic patterns.

**Results:**

Particularly the Cox model is prone to potentially severe *selection bias* and *direct effect bias*, resulting in underestimation when restricting the analysis to first events. We find that both kinds of bias can simultaneously be reduced by adequately incorporating recurrent events into the analysis model. Correspondingly, we point out appropriate proportional hazards-based multi-state models for decreasing bias and increasing power when analyzing multiple-episode composite endpoints in randomized clinical trials.

**Conclusions:**

Incorporating multiple episodes per individual into the primary analysis can reduce the bias of a treatment’s total effect estimate. Our findings will help to move beyond the paradigm of considering first events only for approaches that use more information from the trial and augment interpretability, as has been called for in cardiovascular research.

## Background

When analyzing composite endpoints that incorporate an endpoint with multiple episodes, such as hospital admission, a time to first event approach is frequently adopted for randomized clinical trials. Researchers from different disciplines have called for more appropriate methods of statistical analysis to more closely reflect the patients’ disease burden. This involves a discussion on whether multiple episodes per patient are to be analyzed. So far, this discussion mostly has considered power issues, while overlooking potential bias. In this work, we investigate sources of bias and show that there can be a potentially severe underestimation of treatment effect estimates, when derived only based on first events, that can be substantially reduced by adequately modeling multiple episodes per patient.

Composite endpoints combine several events of interest into a single variable, usually defined as a time to event outcome. They are frequently used as primary or secondary endpoints in cardiovascular clinical trials [1, 2]. Composite outcomes facilitate the evaluation of treatment effects when unrealistically large sample sizes would be required to detect differences in the incidence of single outcomes among treatment groups, for example mortality. While using a composite outcome may help in terms of power, at the same time it introduces its own difficulties concerning interpretation of trial results and methodological challenges [2–6]. One major concern is that endpoints occurring in individual patients usually are clinically related (such as nonfatal and fatal myocardial infarctions). Multi-state modeling of these relations by allowing for separate transition hazards between the different subsequent events has recently been proposed for large cardiovascular observational studies [7, 8]. However, for randomized clinical trials this is suspected to attenuate the power and confirmatory character of the trial [9]. In the majority of clinical trials, the concern for potential relations between clinical episodes is therefore addressed by counting only one event per patient and analyzing the time to the first of all components. By following this approach, only data on the first episode per individual are used for the primary statistical analysis, even when subsequent episodes (including deaths) have been recorded. There is an ongoing debate, in particular in cardiovascular research, on the efficiency and validity of this practice because it ignores a great deal of clinically relevant information [3, 10–12]. The impact of multiple episodes per patient on the power of a clinical trial is apparently promising [3, 13], and selected statistical methods have been exemplarily applied to single trial data [14–16]. However, less attention is paid to the estimation and interpretability of treatment effects that can be substantially attenuated depending on whether multiple episodes are analyzed or not. We consider this critical since the choice of a statistical method for analyzing trial data should not be mainly driven by power considerations but by the objective to obtain an unbiased and meaningful treatment effect estimate, i.e. to make causal inferences about the treatment and its (added) benefit and to understand how a treatment influences a patient’s disease burden.

Although randomized clinical trials are often suspected to produce unbiased results as the randomized treatment allocation prevents confounding, hazard-based survival analysis can introduce its own bias [17–20]. In particular, the Andersen-Gill approach [21] has been suspected to introduce bias by erroneously modeling that a clinical episode will leave a patient’s risk profile unchanged and will not affect the incidence rate for future episodes [22–25]. This finding has been controversially discussed as it implicitly assumes that direct effects are to be estimated [26]. The causal directed acyclic graphs approach (DAG) [27, 28] has been proposed for defining adequate statistical models that prevent or minimize bias in the presence of confounding. It is a powerful tool for identifying and addressing bias and is increasingly popular, but it is primarily applied in epidemiological research. In this work, we will make use of this approach for randomized clinical trials to provide an accessible explanation of potential bias in proportional hazards-based survival analysis of first and multiple episodes of a composite endpoint and to define adequate statistical models for reducing or preventing bias. While the use of DAGs may be problematic in a continuous time setting [29], we are avoiding such issues by first considering actual discrete states in DAG analysis, and making the transition to continuous time settings with evidence from simulations.

The article is organized as follows: We motivate this research with a clinical example in “Cardiovascular clinical trial example” section. Then, in “Methods” section, we first formalize potential bias via directed acyclic graphs and illustrate the findings on simplified examples. Thereafter we identify statistical models that have the potential to reduce that bias. We support our findings by simulation studies that mimic the motivating clinical trial situation and present the results in “Results” section. Finally, we finish the article with a discussion in “Discussion” section.

## Cardiovascular clinical trial example

This work has been motivated by the ST2 guided tReatment upON discharGe in Heart Failure (STRONG-HF) trial, a randomized controlled clinical trial that has been planned to investigate whether heart failure patients will benefit from a biomarker-based treatment scheme compared to standard care. It is planned as a multicenter prospective, randomized, open-label for patients, blinded-endpoint and event-driven study. The primary endpoint was defined as a composite of cardiovascular mortality and recurrent worsening heart failure. Worsening heart failure includes hospitalization due to heart failure or urgent visit to the emergency department or heart failure clinic due to decompensation needing unplanned intravenous diuretic treatment. Patients are to be uniformly recruited over a period of one year and are to be followed for one year after the end of the recruitment phase. The two regimens are to be allocated randomly and in a balanced fashion among the recruited patients. In addition to the treatments’ effect on the combined endpoint, its effects on the single components, cardiovascular death and disease-associated admissions, are also of major interest. From previous data, an annual death rate of 0.14 and an annual admission rate of 1.17 is expected for the patients under standard care (control group), defining a hazard rate for the composite endpoint of *λ*=1.31. Treatment is expected to decrease that rate by 25%, corresponding to a hazard ratio of HR =0.75. When the time to first composite endpoint is analyzed, a total number of *N*=465 patients is required to attain a power of 80% for rejecting the null hypothesis of no treatment effect on the incidence of the composite endpoint *H*_0_={*H* *R*=1} [30]. Incorporating recurrent events into the statistical analysis has the potential to decrease the sample size to up to *N*=223 [13], and thus is apparently promising for improving the feasibility and efficiency of the trial. However, disease-associated complications that require a hospital admission will obviously affect the risk for further non-fatal and fatal outcomes. For example, patients who acquire a non-fatal MI have an increased risk for fatal and non-fatal outcomes thereafter. Concern arises if this might question the study results, and, more generally, how incorporating recurrent events into the primary statistical analysis will affect the treatment effect estimates and thus the interpretation of trial results.

## Methods

### Formalizing potential bias via directed acyclic graphs

The graphical representation of causal effects between variables [27, 28] helps to understand the sources of potential bias when estimating some causal effect of an exposure to an outcome and how different statistical models differently address that bias. In the causal directed acyclic graph (DAG) approach, an arrow connecting two variables indicates causation; variables with no direct causal association are left unconnected. We will use this approach for illustrating the causal system in randomized clinical trials when a composite endpoint is investigated that comprises fatal and non-fatal events. An example is the composite of cardiovascular death and hospital admission for heart failure disease as defined in the motivating clinical trial example (“Cardiovascular clinical trial example” section). Effect estimation is assumed to be hazard-based with a proportional hazards assumption.

#### Selection bias

**Fig. 1:**
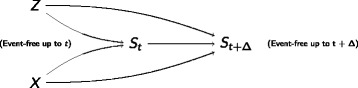
Directed acyclic graph for the causal system between treatment (*X*), being free of any event at time *t* (*S*_*t*_) and *t*+*Δ* (*S*_*t*+*Δ*_), and unobserved variables (Z) that are unrelated to treatment (for example by randomization) and affect the event rate. Figure according to Aalen et al. [20]

Figure 1 illustrates the causal system in a time to first composite endpoint approach. The randomized treatment (*X*) is the exposure variable, that is assumed to affect the fatal and non-fatal outcomes and thus the composite endpoint. In addition to treatment, further disease or patient characteristics will affect the risk for adverse outcomes. Some are known, others are unknown or unmeasurable (summarized as a single unobserved variable Z). Obviously, being free of any event at time *t* (*S*_*t*_) is a collider on the path between the exposure treatment and the unobserved variable Z. Conditioning on a collider will open the path between the variables that are connected by the collider and thus artificially introduce spurious associations [31]. Each contribution to the partial likelihood in the Cox proportional hazards model is a conditional contribution, conditional on being free of any event up to that time. Therefore, an association is induced between the actually unrelated randomized treatment and the unobserved variable Z. As Z affects the outcomes, this association will bias the treatment’s effect estimate for the fatal, non-fatal and composite outcome. This bias is called *selection bias* and has been investigated for incidence rate ratios [28] and hazard ratios [17, 18, 20, 32] before. We will illustrate selection bias by a simple example at the end of this subsection. Whereas conditioning on being alive is an unavoidable step in the hazard-based analysis, we can prevent conditioning on being free of any event by including the recurrent non-fatal events into the statistical model: the at-risk set in the partial likelihood estimator then comprises all subjects that are still alive in contrast to a set of those subjects only that are free of any event at the particular time point. This way, incorporating recurrent events will reduce selection bias when estimating the treatment effect on the fatal and non-fatal outcomes and will thus also reduce the bias when estimating the treatment effect on the composite endpoint. In summary, the first insight gained from a formalization via DAGs is that analyzing all non-fatal events, also the recurrent ones, in the statistical model for the composite endpoint will reduce selection bias.

##### Example

Consider a balanced randomized trial comparing the time to first event under a particular treatment, as compared to some control intervention. Further assume that the study population consists of two equally-sized subgroups, a low-risk group and a high-risk group, specified by an unobserved variable *Z* (Fig. 1). For illustrating selection bias we consider a setting with discrete times (which can be readily transferred to the continuous time Cox proportional hazards model [33]) with failures occurring only at times *t*_1_ and *t*_2_. In the control group the risk for experiencing an event at time *t*_1_ is assumed to be 1/3 in the low-risk-group and 2/3 in the high-risk-group, respectively. The same risk probabilities are assumed for experiencing an event at time *t*_2_ in the subset of subjects that are still at risk before *t*_2_, i.e. having not experienced an event at *t*_1_. The odds ratio for treatment compared to control, which is the discrete-time equivalent to the continuous time hazard ratio [33], is assumed to be 1/2 within each subgroup and for each time *t*_1_ and *t*_2_ (constant hazard ratio assumption). From the odds ratio and the expected failure rates per subgroup in the control group at *t*_1_, we can derive the expected failure rates at *t*_1_ for the treatment group, which are 1/5 and 1/2, respectively. For the example of a sample size of *N*=1800 per treatment group, Table 1 shows the number of event-free subjects just before *t*_2_ per treatment group and subgroup and the expected number of failures at *t*_2_ as derived from the expected failure rates of 1/3 and 2/3 in the control group and 1/5 and 1/2 in the treatment group. Whereas the odds ratio is unbiased when estimated within each subgroup (0.5), the crude odds ratio estimated from the marginal table is 0.57, indicating a smaller treatment effect (selection bias) that is obtained when conditioning on being event-free but not taking *Z* into account. As indicated, *Z* might be unobserved, making conditioning on *Z* problematic. The selection bias does not depend on sample size, which was chosen to be large in this data example to obtain integer patient numbers. The difference between conditional and unconditional modeling remains when moving from discrete time to continuous time, i.e. when the interval between two potential failures becomes infinitesimally small and the hazard ratio is defined on a continuous time scale. Simulation results (“Simulation studies” section) will further support this finding.

**Table 1:** Expected patient numbers in the discrete failure time example for time to first event stratified by subgroup (“Selection bias” section)

Stratum	Group	Event at *t*_2_	No event at *t*_2_	At risk at *t*_2_	OR
Low-risk subgroup	Treatment	144	576	720	0.5
	Placebo	200	400	600	
High-risk subgroup	Treatment	225	225	450	0.5
	Placebo	200	100	300	
All patients (unstratified)	Treatment	369	801	1170	0.57
	Placebo	400	500	900	

Patients are at risk for a first event at *t*_2_, if they have not experienced an event at *t*_1_. Odds Ratio (OR) for experiencing an event under treatment as compared to control

#### Direct effect bias

**Fig. 2:**
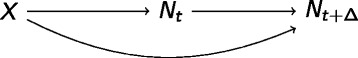
Directed acyclic graph for the causal system between treatment (*X*) and the number of events up to time *t* (*N*_*t*_) and *t*+*Δ* (*N*_*t*+*Δ*_)

When following the recommendation to include recurrent events into the statistical model as derived from the previous section, concern might arise as to how to model the transitions from one non-fatal event to a succeeding fatal or non-fatal event. From cardiovascular research it is well known that the different components in a composite endpoint are related. For example, a non-fatal myocardial infarction will apparently affect the risk for further fatal or non-fatal cardiovascular outcomes. To address this concern, we will again apply the approach of directed acyclic graphs. Figure 2 illustrates the causal system when more than only the first event is considered and the risk for further events is potentially changing with each non-fatal event. The number of events experienced until time *t*, *N*(*t*), is a mediator lying along the causal pathway between treatment *X* and the number of events at *t*+*Δ*, *N*(*t*+*Δ*). Conditioning on or stratifying by the number of previously experienced events will close this path, and the treatment effect estimate is reduced to the treatments’ direct effect on the outcome, whereas its indirect effect is not considered. While direct effects are interesting from a biological viewpoint, estimation of total effects is important from the clinical, health care, and patients’ perspective. For example, the mortality rate increases after a non-fatal myocardial infarction, and therefore a treatment that effectively prevents myocardial infarctions in general reduces the mortality (indirect effect), besides its direct effect on mortality. Both, direct and indirect effects, define a treatment’s total effect. We will illustrate the difference between direct and total effect estimation in a simple example at the end of this section. In summary, the second insight gained from a formalization via DAGs is to not condition on the individual’s event history by stratifying or adjusting for the previous non-fatal events for estimating a treatments total effect. In contrast, in a time to first event analysis, the effect estimate is naturally restricted to the direct effect as it is derived only from those pathways, that start from the exposure variable treatment. We use the term *direct effect bias* when effect estimates are reduced to direct effects only.

##### Example

Again, consider a balanced randomized trial comparing the time to event under a particular treatment as compared to some control intervention. As before, we consider a setting with discrete times, that can be transferred to the continuous time Cox proportional hazards model. We assume that non-fatal events are experienced at time *t*_1_ and can be followed by death at time *t*_2_. The risk for experiencing a non-fatal event at *t*_1_ is assumed to be 2/3 in the control group and 1/3 in the treament group, respectively. The mortality rate in patients who have acquired the non-fatal event at *t*_1_ increases to 40% as compared to a 20% risk in those subjects who are free of an event at *t*_1_. Mortality rates are assumed to be not affected by treatment conditionally on the number of prior events, i.e. neither before nor after having experienced a non-fatal event. For the example of a total sample size of *N*=1800, Table 2 shows the expected number of death cases stratified by having experienced a preceding non-fatal event at *t*_1_ and marginally over all subjects. Whereas within each stratum no treatment effect on mortality is observed, respectively, the odds ratio estimated from the marginal table is 0.73, indicating a positive treatment effect on mortality. This result indicates that treatment effectively reduces mortality by preventing subjects from entering that stratum, which is characterized by a higher mortality rate (total effect), although it has no direct effect on the mortality rates at all. Effect estimates differ when conditioning on prior events or not, irrespective of sample size, and when moving from discrete time to continuous time, thus when deriving the hazard ratio in a continuous time scale. Simulation results (“Simulation studies” section) will further support this finding.

**Table 2:** Expected patient numbers in the discrete failure time example for time to death stratified by previously experienced non-fatal event (“Direct effect bias”section)

Stratum	Group	Death at *t*_2_	Alive at *t*_2_	At risk	OR
Non-fatal event at *t*_1_	Treatment	120	180	300	1
	Placebo	240	360	600	
No non-fatal event at *t*_1_	Treatment	120	480	600	1
	Placebo	60	240	300	
All patients (unstratified)	Treatment	240	660	900	0.73
	Placebo	300	600	900	

Odds Ratio (OR) for mortality under treatment as compared to control

### Reducing bias by statistical modeling

We will now transfer the insights on biased effect estimation as derived from the DAGs to identify statistical analysis models that have the potential to reduce that bias. Consider a randomized clinical trial with *n* subjects followed for a composite endpoint. Subjects will be indexed by *i*, events by *j*. Let $T_{CE,ij}^{*}$ be a series of random variables that describe the time from starting point 0 to the *j*-th occurrence of the composite endpoint in subject *i*. Let further *C*_*i*_ be independent identically distributed random variables that describe the time to censoring. We observe $T_{CE,ij}=min(T_{CE,ij}^{*},C_{i})$, the time to composite endpoint or censoring, whichever comes first, and the indicator variables $\delta _{ij}=\textbf {I}\left \{T_{CE,ij}^{*}\leq C_{i}\right \}$.

**Fig. 3:**
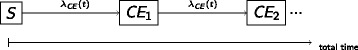
Unstratified transition hazard model for the transitions between study start (*S*) and the recurrent composite endpoints (*C* *E*_1_, *C* *E*_2_…)

It has been proposed to describe the distribution of *T*_*CE*,*ij*_ by a multiplicative intensity process [34], *Y*_*i*_(*t*)·*λ*_*CE*,*i*_(*t*), of the underlying counting process

$$N_{i}(t):=\#\left\{j; \;T_{CE,ij} \leq t \;\wedge\; T^{*}_{CE,ij} \leq C_{i}\right\}, $$

with deterministic hazard function *λ*_*CE*,*i*_(*t*) (Fig. 3) and *Y*_*i*_(*t*)=**I**{*t*≤*C*_*i*_}. Figure 3 sketches a model that comprises all events, also the recurrent ones (*C* *E*_1_, *C* *E*_2_…), without conditioning on or stratifying by the event history (transition hazards between the succeeding events do not change). If conditional on covariates *λ*_*CE*,*i*_ has a Cox proportional hazards shape, this model is known as the Andersen-Gill [21] model.

1 $$ \lambda_{CE}(t|X_{i}) = \lambda_{CE,0}(t) \cdot \exp(\beta X_{i}')  $$

with *X*_*i*_ being the *p*-dimensional vector of covariates for subject *i* and *β* being the vector of regression coefficients. The Andersen-Gill model was recently applied to re-analyze clinical trials in patients suffering from heart failure to evaluate the effect of new therapies on the patients risk of the composite of hospitalizations due to heart failure and cardiovascular death [14–16]. The treatment effect *β* is then estimated by maximizing the partial likelihood

2 $$\begin{array}{@{}rcl@{}} PL^{AG}(\beta) &=& \prod_{\mathrm{i}} \prod_{\mathrm{j}} \left(\frac{\exp(\beta X_{i}')}{{\sum\nolimits}_{k \in R_{(ij)}^{AG}}\exp(\beta X_{k}')} \right)^{\delta_{ij}} \end{array} $$

The at-risk set $R_{(ij)}^{AG}$ includes all subjects who have not been censored and have not died before time *t*_*ij*_, the time when individual *i* experiences its *j*-th event. In contrast, in a stratified model as proposed by Prentice et al. [35], the at-risk set $R_{(ij)}^{PWP}$ is restricted to only those subjects who are at risk for experiencing the *j*-th event at time *t*_*ij*_, thus having experienced *j*−1 events before. However, following the arguments of “Formalizing potential bias via directed acyclic graphs” section, the Andersen-Gill model allows the estimation of total effects by not stratifying on the event history, in contrast to the stratified model that is estimating direct effects only [26]. Both models are still susceptible to selection bias as they naturally restrict the risk sets to subjects being alive. However, they reduce the selection bias as compared to results derived from a Cox proportional hazards model with partial likelihood

3 $$\begin{array}{@{}rcl@{}} PL^{C}(\beta) &=& \prod_{\mathrm{i}} \left(\frac{\exp(\beta X_{i}')}{{\sum\nolimits}_{k \in R_{(i)}^{C}}\exp(\beta X_{k}')}\right)^{\delta_{i1}} \end{array} $$

as in this model the risk sets $R_{(i)}^{C}$ are restricted to subjects that are not only still alive but also free of any previous non-fatal event at time *t*_*i*1_, the time of the first event or censoring of individual *i*.

**Fig. 4:**
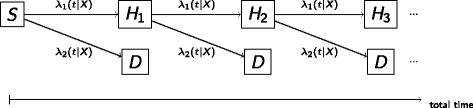
Multi-state model for the transitions between study start (*S*), recurrent hospital admissions (*H*_*i*_) and death (*D*) stratified by the event type but un-stratified by the number of preceeding hospital admissions

The partial likelihood (2) of the unstratified maximally unrestricted Andersen-Gill model (1) can be re-written as

4 $$\begin{array}{@{}rcl@{}} PL^{AG}(\beta) &=& \prod_{l}\prod_{i} \prod_{j_{l}} \left(\frac{\exp(\beta X_{i}')}{{\sum\nolimits}_{k \in R_{(ij_{l})}^{AG}}\exp(\beta X_{k}')}\right)^{\delta_{ij_{l}}} \end{array} $$

with *l*=1,…,*L* indexing the *L* components of the composite and *j*_*l*_ indexing the events of type *l* and $\delta _{ij_{l}}$ again being the corresponding event indicator. Therefore, model (2) can also be described as a multi-state model that allows for different baseline transition hazards for the different components. Figure 4 sketches this model for the motivating example of two components: death (*D*) and hospital admission (*H*_1_,*H*_2_…) with

5 $$\begin{array}{@{}rcl@{}} \lambda_{l}(t|X_{i})&=& \lambda_{l,0}(t)\exp(\beta X_{i}'), l=1,2. \end{array} $$

By defining a single vector *β* for both the transition hazards *λ*_1_ and *λ*_2_, a constraint is induced, namely that the covariates equally affect fatal and non-fatal events. In particular, for our motivating example this means that treatment has the same effect on the fatal and non-fatal outcomes. This constraint has in fact been described as a requirement for the proper use of composite endpoints, for example by regulatory agencies [36]. However, at the same time it has been observed that in practice this assumption is frequently violated. Ferreira-Gonzalez et al. [37] conclude from a systematic literature review that effects of treatments in cardiovascular clinical trials differ strongly between the components, with larger effects in less relevant components and the smallest effects in mortality. The same has been observed in several clinical trials on heart failure disease [14, 16]. To relax the constraint of a common treatment effect on all components, the more general multi-state model (MS) can be defined by transition hazards

6 $$\begin{array}{@{}rcl@{}} \lambda_{l}(t|X_{i})&=& \lambda_{l,0}(t)\exp(\beta_{l} X_{i}'), l=1\ldots{L} \end{array} $$

and partial likelihood

7 $$\begin{array}{@{}rcl@{}} {}PL^{MS}(\beta_{1}, \ldots,\beta_{L}) &\,=\,& \!\prod_{l}\prod_{i} \prod_{j_{l}}\! \left(\!\frac{\exp(\beta_{l} X_{i}')}{{\sum\nolimits}_{k \in R_{(ij_{l})}^{MS}}\exp(\beta_{l} X_{k}')}\!\right)^{\delta_{ij_{l}}}. \end{array} $$

and risk sets $R_{(ij_{l})}^{MS}$ that include all subjects who have not been censored and have not died before the particular event time, respectively. This generalization of the Andersen-Gill model allowing for separate treatment effects for each component, *β*_*l*_, can be proposed whenever sample size and event frequency allow for such an approach. It still does not stratify on the event history and does not restrict the at-risk-set only to those subjects that are free of any event, but allows for a higher flexibility with respect to differential treatment effects.

Note, that we focus on marginal models within this manuscript. By introducing a (joint) frailty term into model (5) or (6) and applying penalized likelihoods [38], a conditional joint frailty model could also be fitted. By conditioning on the frailty term the selection bias as illustrated in Fig. 1 is minimized, however at the price of increasing the model complexity by introducing further model assumptions (joint frailty distribution) and parameters (frailty variance). We will show in the next section that in many applications one can safely stay with the marginal model, thereby following the *Occam’s razor principle*.

### Simulation studies

We investigate the bias in treatment effect estimation as identified in “Formalizing potential bias via directed acyclic graphs” section (selection bias, direct effect bias) in simulation studies. The simulation study mimics the clinical trial situation that has motivated this research. For this purpose, we consider a balanced randomized clinical trial with a follow-up of two years and uniformly distributed recruitment of *N*=380 individuals over the first year. The transition hazards *λ*_1_ and *λ*_2_ (Fig. 4 and Eq. (6)) for the transitions to fatal and non-fatal events, respectively, are defined by *λ*_*l*_(*t*|*X*_*i*_)=*λ*_*l*_· exp(*β*_*l*_ *X*_*i*_).

Baseline annual death and admission rates are defined as *λ*_1_=0.14 and *λ*_2_=1.17, respectively. Further simulations are performed where fatal and non-fatal events equally contribute to the same overall annual event rate, thus *λ*_1_=*λ*_2_=0.655. Treatment is assumed to equally affect both components of the composite, and we define *β*=*β*_1_=*β*_2_= log(0.75) as was expected in the planning phase of the STRONG-HF trial. We additionally consider situations where treatment has a minor effect on mortality (*β*_1_= log(0.92)), following the findings of a systematic literature review on cardiovascular clinical trials [37]. To consider that unobserved or unmeasurable variables affect the outcomes, we define an unobserved variable *Z*_*i*_ per individual *i*. The *Z*_*i*_ are generated as independent and gamma-distributed random variables with mean 1 and variance *θ*. Following a frailty approach, *Z*_*i*_ is assumed to act multiplicatively on the hazard by

8 $$\begin{array}{@{}rcl@{}} \lambda_{l}(t|X_{i},Z_{i})&=& \lambda_{l,0}(t)\cdot Z_{i} \cdot \exp(\beta_{l} X_{i}), l=1,2 \end{array} $$

Note that the unobserved variable acts on both transition hazards, inducing a correlation between both processes. Such a joint model [38] is considered to more closely mimic real clinical trial data as compared to simulation models assuming independency between the event processes, as in most situations it can be expected that patient and disease characteristics will affect adverse disease outcomes towards the same direction. Different *θ*∈{0,0.2,…,1} reflect different strengths of association between the unobserved variable and the fatal and non-fatal outcomes and will therefore cause different degrees of selection bias. In a second simulation study we add an indirect effect of treatment on the composite outcome by defining the transition hazards to be increasing by a factor of *ρ* with each non-fatal event. By applying a range of values between *ρ*=1 (no increase of hazards) and *ρ*=1.3 (increase of hazards by 30% with each non-fatal event), different degrees of the indirect effects are evaluated.

In a third simulation study we investigate treatment effect estimation when both effects are present, that is the transition hazards increase with each non-fatal events by a factor of *ρ* (*ρ*∈ [ 1,1.3]) while in addition a gamma-distributed frailty term with mean 1 and a moderate variance of *θ*=0.6 acts on all transition hazards. For each simulation model 5000 datasets are simulated, respectively.

All simulated data are analyzed by the Andersen-Gill model for the composite endpoint (1) and its multi-state extension (6) to estimate separate treatment effects on fatal and non-fatal outcomes. Both models are applied to the full simulated datasets and to datasets that are restricted to the first composite endpoint per individual. For the restricted data, the Andersen-Gill model then reduces to a Cox proportional hazards model and its multi-state extension to a competing risk model.

All data are simulated and analyzed in the open-source statistical environment R, version 3.1.0 (2014-04-10) [39] and by extending the published simulation algorithm for recurrent event data [40]. Mean regression coefficient estimates are derived together with standard errors as estimated from their variability among the simulations.

## Results

**Fig. 5:**
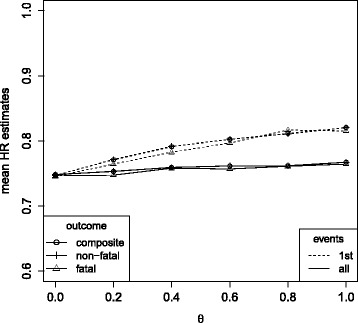
Mean hazard ratio estimates in the simulation model under *λ*_1_=0.14,*λ*_2_=1.17, *ρ*=1, a common treatment effect on non-fatal and fatal outcomes (*β*_1_=*β*_2_= log(0.75)) and varying influence of an unobserved variable *Z* (having variance *θ*). Cox proportional hazards analysis for the composite and the single components, respectively (1st events), Andersen-Gill analysis (all events, composite outcome) and multi-state analysis (all events, fatal and non-fatal outcomes)

**Fig. 6:**
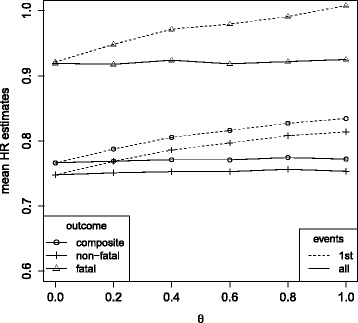
Mean hazard ratio estimates in the simulation model under *λ*_1_=0.14,*λ*_2_=1.17, *ρ*=1, a lower treatment effect on fatal than on non-fatal outcomes (log(0.92)=*β*_1_>*β*_2_= log(0.75)) and varying influence of an unobserved variable *Z* (having variance *θ*). Cox proportional hazards analysis for the composite and the single components, respectively (1st events), Andersen-Gill analysis (all events, composite outcome) and multi-state analysis (all events, fatal and non-fatal outcomes)

Simulation results are presented in Tables 3 and 4 for *λ*_1_∈{0.14,0.655}, *β*_1_∈{log(0.92), log(0.75)}, *ρ*∈{1,1.05,1.1,1.15,1.2,1.25,1.3} and *θ*∈{0,0.6}. In addition, Fig. 5 summarizes the simulation results for data following model (8), where transition hazards are equally affected by treatment and unaffected by non-fatal events (*ρ*=1), but a common unobserved variable *Z* acts multiplicatively on each transition hazard. Mean treatment regression coefficient estimates are given dependent on the variance of *Z* (*θ*) when applying the Cox proportional hazards analysis for the time to first event to each particular outcome (1st events), when applying the Andersen-Gill modeling approach (1) for the time to recurrent composite endpoints (all events, composite outcome) and when applying the multi-state modeling approach (6) to the recurrent events (all events, fatal and non-fatal outcome). The extent of bias that is introduced by conditioning on being event-free (1st event analyses) is increasing with the strength of association between the unobserved variable and the fatal and non-fatal outcomes, supporting the findings of “Formalizing potential bias via directed acyclic graphs” section. The statistical analysis models incorporating recurrent events do not condition on being event-free and thus substantially decrease the selection bias. The bias that is still remaining is only small, because it is caused by conditioning on survival status and the mortality rate was assumed to be low as observed in most cardiovascular trials [37]. When the mortality rate has a larger contribution to the overall event rate of *λ*_1_+*λ*_2_=1.31 (*λ*_1_=0.655), selection bias in the analysis of recurrent events slightly increases as compared to the situation with a mortality rate of only *λ*_1_=0.14. The higher the mortality rate, the more conditioning on being alive is affecting the partial likelihood estimates, which explains this result. However, bias remains small ($\exp (\hat {\beta _{1}})=\exp (\hat {\beta _{2}})=0.78$ when exp(*β*_1_)= exp(*β*_2_)=0.75 and $\exp (\hat {\beta _{1}})=0.93$, $\exp (\hat {\beta _{2}})=0.76$ when exp(*β*_1_)=0.92 and exp(*β*_2_)=0.75) for *θ*=0.6 and *ρ*=1 (Table 4). When treatment differentially affects the risk for fatal and non-fatal outcomes (Fig. 6), the treatment regression coefficient

**Table 3:** Simulation results for *λ*_1_=0.14 and *λ*_2_=1.17

Simulation parameters					Results of 1st-event-analyses							Results of all-events-analyses					
exp(*β*_1_)	exp(*β*_2_)	*θ*	*ρ*		$\exp (\widehat {\beta _{CE}})$	$\widehat {SE}(\beta _{CE})$	$\exp (\widehat {\beta _{1}})$	$\widehat {SE}(\beta _{1})$	$\exp (\widehat {\beta _{2}})$	$\widehat {SE}(\beta _{2})$		$\exp (\widehat {\beta _{CE}})$	$\widehat {SE}(\beta _{CE})$	$\exp (\widehat {\beta _{1}})$	$\widehat {SE}(\beta _{1})$	$\exp (\widehat {\beta _{2}})$	$\widehat {SE}(\beta _{2})$
0.75	0.75	0.00	1.00		0.75	0.11	0.75	0.37	0.75	0.12		0.75	0.08	0.75	0.26	0.75	0.09
0.75	0.75	0.00	1.05		0.75	0.11	0.74	0.37	0.75	0.12		0.74	0.08	0.73	0.25	0.74	0.09
0.75	0.75	0.00	1.10		0.75	0.12	0.75	0.37	0.75	0.12		0.73	0.09	0.73	0.25	0.73	0.09
0.75	0.75	0.00	1.15		0.75	0.12	0.76	0.37	0.75	0.12		0.72	0.09	0.72	0.24	0.72	0.10
0.75	0.75	0.00	1.20		0.75	0.12	0.75	0.37	0.75	0.12		0.70	0.09	0.70	0.24	0.70	0.10
0.75	0.75	0.00	1.25		0.75	0.11	0.74	0.37	0.75	0.12		0.68	0.10	0.68	0.23	0.68	0.11
0.75	0.75	0.00	1.30		0.75	0.12	0.74	0.37	0.75	0.12		0.65	0.12	0.65	0.22	0.65	0.13
0.75	0.75	0.60	1.00		0.80	0.13	0.80	0.40	0.80	0.13		0.76	0.12	0.76	0.26	0.76	0.12
0.75	0.75	0.60	1.05		0.80	0.13	0.80	0.40	0.80	0.13		0.75	0.12	0.75	0.26	0.75	0.13
0.75	0.75	0.60	1.10		0.80	0.13	0.80	0.41	0.80	0.13		0.74	0.13	0.74	0.26	0.74	0.13
0.75	0.75	0.60	1.15		0.80	0.12	0.80	0.40	0.80	0.13		0.73	0.13	0.72	0.24	0.73	0.14
0.75	0.75	0.60	1.20		0.80	0.13	0.80	0.41	0.81	0.13		0.73	0.14	0.71	0.24	0.73	0.15
0.75	0.75	0.60	1.25		0.81	0.13	0.80	0.40	0.81	0.13		0.72	0.15	0.70	0.23	0.73	0.15
0.75	0.75	0.60	1.30		0.81	0.13	0.80	0.41	0.81	0.13		0.72	0.15	0.69	0.22	0.72	0.16
0.92	0.75	0.00	1.00		0.77	0.12	0.92	0.35	0.75	0.13		0.77	0.08	0.92	0.24	0.75	0.09
0.92	0.75	0.00	1.05		0.77	0.11	0.92	0.35	0.75	0.12		0.76	0.08	0.90	0.24	0.74	0.09
0.92	0.75	0.00	1.10		0.77	0.12	0.92	0.35	0.75	0.12		0.75	0.09	0.90	0.23	0.73	0.09
0.92	0.75	0.00	1.15		0.77	0.12	0.93	0.34	0.75	0.12		0.74	0.09	0.88	0.23	0.72	0.10
0.92	0.75	0.00	1.20		0.77	0.11	0.92	0.35	0.75	0.12		0.72	0.09	0.86	0.22	0.70	0.10
0.92	0.75	0.00	1.25		0.77	0.12	0.93	0.35	0.75	0.12		0.70	0.11	0.83	0.22	0.68	0.11
0.92	0.75	0.00	1.30		0.77	0.12	0.92	0.35	0.75	0.12		0.66	0.12	0.79	0.21	0.65	0.12
0.92	0.75	0.60	1.00		0.82	0.12	0.98	0.38	0.80	0.13		0.77	0.11	0.92	0.25	0.75	0.12
0.92	0.75	0.60	1.05		0.82	0.12	0.98	0.38	0.80	0.13		0.76	0.12	0.91	0.24	0.74	0.13
0.92	0.75	0.60	1.10		0.82	0.13	0.98	0.38	0.80	0.13		0.75	0.12	0.89	0.24	0.73	0.13
0.92	0.75	0.60	1.15		0.82	0.12	0.98	0.39	0.80	0.13		0.74	0.13	0.87	0.23	0.72	0.14
0.92	0.75	0.60	1.20		0.82	0.13	0.98	0.38	0.80	0.13		0.73	0.14	0.85	0.22	0.71	0.15
0.92	0.75	0.60	1.25		0.82	0.12	0.97	0.38	0.80	0.13		0.72	0.15	0.82	0.21	0.71	0.15
0.92	0.75	0.60	1.30		0.82	0.13	0.98	0.38	0.80	0.14		0.72	0.15	0.81	0.21	0.70	0.16

**Table 4:** Simulation results for *λ*_1_=0.655 and *λ*_2_=0.655

Simulation parameters					Results of 1st-event-analyses							Results of all-events-analyses					
exp(*β*_1_)	exp(*β*_2_)	*θ*	*ρ*		$\exp (\widehat {\beta _{CE}})$	$\widehat {SE}(\beta _{CE})$	$\exp (\widehat {\beta _{1}})$	$\widehat {SE}(\beta _{1})$	$\exp (\widehat {\beta _{2}})$	$\widehat {SE}(\beta _{2})$		$\exp (\widehat {\beta _{CE}})$	$\widehat {SE}(\beta _{CE})$	$\exp (\widehat {\beta _{1}})$	$\widehat {SE}(\beta _{1})$	$\exp (\widehat {\beta _{2}})$	$\widehat {SE}(\beta _{2})$
0.75	0.75	0.00	1.00		0.75	0.12	0.75	0.17	0.75	0.16		0.75	0.10	0.75	0.14	0.75	0.14
0.75	0.75	0.00	1.05		0.75	0.11	0.75	0.16	0.75	0.16		0.74	0.10	0.75	0.14	0.74	0.14
0.75	0.75	0.00	1.10		0.75	0.11	0.75	0.16	0.75	0.16		0.74	0.10	0.74	0.14	0.74	0.14
0.75	0.75	0.00	1.15		0.75	0.12	0.75	0.17	0.75	0.16		0.74	0.10	0.74	0.14	0.74	0.14
0.75	0.75	0.00	1.20		0.75	0.12	0.75	0.16	0.75	0.16		0.73	0.10	0.74	0.13	0.73	0.14
0.75	0.75	0.00	1.25		0.75	0.12	0.75	0.17	0.75	0.16		0.73	0.10	0.73	0.14	0.73	0.15
0.75	0.75	0.00	1.30		0.75	0.12	0.75	0.16	0.75	0.17		0.73	0.11	0.73	0.13	0.72	0.15
0.75	0.75	0.60	1.00		0.80	0.12	0.80	0.18	0.80	0.18		0.78	0.12	0.78	0.15	0.78	0.17
0.75	0.75	0.60	1.05		0.80	0.12	0.80	0.18	0.80	0.18		0.78	0.12	0.78	0.15	0.78	0.18
0.75	0.75	0.60	1.10		0.80	0.12	0.80	0.18	0.80	0.18		0.78	0.12	0.78	0.14	0.78	0.17
0.75	0.75	0.60	1.15		0.80	0.12	0.80	0.18	0.80	0.18		0.78	0.12	0.78	0.15	0.78	0.18
0.75	0.75	0.60	1.20		0.80	0.12	0.81	0.18	0.80	0.18		0.78	0.13	0.78	0.14	0.78	0.18
0.75	0.75	0.60	1.25		0.80	0.13	0.80	0.18	0.80	0.18		0.78	0.13	0.78	0.15	0.78	0.18
0.75	0.75	0.60	1.30		0.80	0.13	0.80	0.18	0.80	0.18		0.78	0.13	0.78	0.14	0.78	0.18
0.92	0.75	0.00	1.00		0.84	0.11	0.92	0.16	0.75	0.17		0.83	0.10	0.92	0.13	0.75	0.14
0.92	0.75	0.00	1.05		0.83	0.12	0.92	0.16	0.75	0.17		0.83	0.10	0.91	0.13	0.75	0.14
0.92	0.75	0.00	1.10		0.84	0.11	0.92	0.16	0.75	0.16		0.83	0.10	0.91	0.13	0.74	0.14
0.92	0.75	0.00	1.15		0.84	0.12	0.92	0.16	0.75	0.17		0.82	0.10	0.90	0.13	0.74	0.15
0.92	0.75	0.00	1.20		0.83	0.11	0.92	0.16	0.75	0.17		0.82	0.10	0.90	0.13	0.73	0.15
0.92	0.75	0.00	1.25		0.83	0.12	0.92	0.16	0.75	0.17		0.81	0.10	0.89	0.13	0.73	0.15
0.92	0.75	0.00	1.30		0.83	0.11	0.92	0.16	0.75	0.17		0.81	0.10	0.89	0.13	0.72	0.15
0.92	0.75	0.60	1.00		0.87	0.12	0.95	0.17	0.78	0.18		0.84	0.12	0.93	0.14	0.76	0.17
0.92	0.75	0.60	1.05		0.87	0.12	0.97	0.17	0.78	0.18		0.84	0.12	0.93	0.14	0.76	0.18
0.92	0.75	0.60	1.10		0.87	0.12	0.96	0.17	0.78	0.18		0.84	0.12	0.93	0.14	0.75	0.18
0.92	0.75	0.60	1.15		0.87	0.12	0.96	0.17	0.78	0.18		0.84	0.12	0.92	0.14	0.75	0.18
0.92	0.75	0.60	1.20		0.87	0.12	0.96	0.17	0.78	0.18		0.83	0.12	0.92	0.14	0.75	0.18
0.92	0.75	0.60	1.25		0.87	0.12	0.96	0.17	0.78	0.18		0.83	0.13	0.91	0.14	0.75	0.19
0.92	0.75	0.60	1.30		0.87	0.12	0.96	0.17	0.78	0.18		0.83	0.13	0.91	0.14	0.74	0.18

 estimates differ by outcome. However, compared to the setting with a common treatment effect (Fig. 5), all effect estimates are similarly affected by selection bias with respect to the direction and magnitude of that bias.

**Fig. 7:**
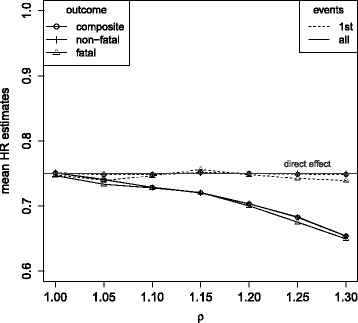
Mean hazard ratio estimates in the simulation model under *λ*_1_=0.14,*λ*_2_=1.17,*θ*=0, a common direct treatment effect on non-fatal and fatal outcomes (*β*_1_=*β*_2_= log(0.75)) and transition hazards that increase by a factor of *ρ* after each non-fatal event. Cox proportional hazards analysis for the composite and the single components, respectively (1st events), Andersen-Gill analysis (all events, composite outcome) and multi-state analysis (all events, fatal and non-fatal outcomes)

Figure 7 shows the simulation results for data randomly generated under transition hazards for fatal and non-fatal events that are equally affected by treatment and increase by a factor of *ρ* with each non-fatal event. No unobserved variable is introduced in this simulation model to clearly differentiate between the different sources of bias. Whereas direct and total effects coincide when transition hazards remain unaffected by previous events (*ρ*=1), Fig. 7 clearly shows that direct and total effects substantially differ when transition hazards increase with non-fatal events (*ρ*>1). The analysis of 1st events provides direct effect estimates whereas the analysis of all events provides total effect estimates according to the findings of “Formalizing potential bias via directed acyclic graphs” section. By preventing experiencing a first non-fatal event, the treatment prevents the patients from becoming at an increased risk for further events. This contributes to the indirect effect, and thus to a larger total treatment effect as compared to its direct effect. Under an increased mortality rate (*λ*_1_=0.655), the process for recurrent events stops earlier on average due to the higher frequency of competing terminal events. Thus, the indirect effect of the treatment (preventing later events that occur with an increased risk rate), contributes less to the total effect estimates. Therefore, differences between total and direct effect estimates become smaller: whereas under *λ*_1_=0.14, *θ*=0 and *ρ*=1.3 the total effect in terms of the hazard ratio is estimated as 0.65 as compared to the direct effect of 0.75 (Table 3), under *λ*_1_=0.655 the total effect estimate of 0.72 is more closely approaching the direct effect (Table 4).

**Fig. 8:**
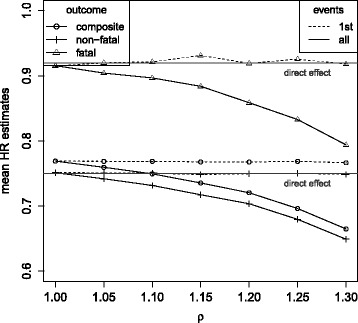
Mean hazard ratio estimates in the simulation model under *λ*_1_=0.14,*λ*_2_=1.17,*θ*=0, a lower direct treatment effect on fatal than on non-fatal outcomes (log(0.92)=*β*_1_>*β*_2_= log(0.75)) and transition hazards that increase by a factor of *ρ* after each non-fatal event. Cox proportional hazards analysis for the composite and the single components, respectively (1st events), Andersen-Gill analysis (all events, composite outcome) and multi-state analysis (all events, fatal and non-fatal outcomes)

Again, when treatment differentially affects the risk for fatal and non-fatal outcomes (Fig. 8), direct and total effect estimates also differ for each single outcome. The direction and magnitude of these differences are comparable to the results observed for common treatment effects (Fig. 7).

As the hazard for the composite endpoint is the sum of the hazards over the two components [41], the hazard ratio can be derived as $1/(\lambda _{1}+\lambda _{2}) {\sum \nolimits }_{i=1}^{2} \lambda _{i} \exp (\beta _{i})$ in the situation of constant hazards. This weighted sum is estimated when analysing the composite outcome using first events only or all events as long as no selection bias and no indirect effects are present, that is *θ*=0 for the analysis of 1st events and *ρ*=1 for the analysis of all events (Figs. 6 and 8). *θ*>0 and/or *ρ*>1 then affect the estimates for the composite endpoint in the same direction as the estimates for the single components.

Whereas selection bias is attenuating the treatment effect estimates, hazards that increase with each non-fatal event induce the total effect estimates to become larger than the direct effect only. As a consequence, the differences between direct and total treatment effect estimates decrease with increasing degree of selection bias. Whereas $\exp (\hat {\beta _{2}})$ decreased from 0.75 to 0.65 when hazards increase by 0 to 30% with each non-fatal event, under *θ*=0.6 only a decrease up to 0.72 is still observed (Table 3). Under a higher mortality rate of *λ*_1_=0.655 even not any decrease in the total effect estimate is observed ($\exp (\hat {\beta _{2}})=0.78$) as here the selection bias starts to prevail (Table 4).

## Discussion

Potential biases in analysis of composite endpoints that comprise endpoints with multiple episodes, such as hospital admission, have been mostly overlooked so far. To advance the state-of-the-art, we provided an accessible explanation of biases in this setting, that is supported by simulation results. Our results show that the initial step in modeling must be defining the treatment effect that is of interest: A total treatment effect estimate can only be derived by analysing all events, whereas only the direct treatment effect can be estimated from analyses of 1st events or from analyses that are stratified by event history. When interpreting trial results, eventually derived from different statistical models, one must be aware, that the direct effect estimates can be severly more prone to selection bias. Our findings will help to move beyond the paradigm of considering first events only for approaches that use more information from the trial and augment interpretability, as has been called for in cardiovascular research [11, 12].

The association of some variable with the outcome is not a reasonable criterion for covariate selection in multiple regression, as has been described in epidemiology for example to explain the birth-weight paradox [42]. We use similar arguments in randomized clinical trials to justify that adjusting or stratifying for the patients’ disease history within trial time is inadequate for estimating a treatments’ total effect.

Selection bias in the Cox proportional hazards model as arising from the non-collapsibility of the hazard ratio estimate [18, 28] has recently been described by Aalen et al. [20]. They use a hypothetical example, where each individual who dies is replaced by an identical individual having the same covariate structure, which would prevent selection bias. In a way, the Andersen-Gill model implements this idea for non-fatal recurrent events by leaving individuals in the risk set after having experienced an event. A terminal component of the composite will still cause selection bias under the Andersen-Gill and multi-state approach. Its magnitude depends on the terminal event rate. Whereas in our simulations, the terminal event rate was small, as observed for most cardiovascular studies [37], and the multi-state models provided nearly unbiased results, Rogers et al. [43] advocate the need for joint frailty models [38, 44] to prevent from bias. However, their findings are based on simulation studies with high mortality rates (up to 31%), which explains these controversial conclusions. Balan et al. [45] recently proposed a score test for deciding between multi-state and joint frailty modeling. All these findings confirm, that using composite endpoints in randomized clinical trials can not eliminate the bias arising from the association between the risk processes of the single components as long as only the first event is analyzed [46].

We have focused on the estimation of a treatment effect based on proportional hazards. Additive hazard models have been recommended instead as they are unaffected by non-collapsibility [20, 47].

Hazard ratios are used to assess the early benefit of new drugs compared to some control [48]. Our results indicate the need to further specify the estimand, the assessment refers to: a treatment’s direct or its total effect as both can differ substantially.

In recent years alternatives to hazard-based analyses of composite endpoints have been proposed based on weighted outcomes [49–51] to consider that not all components are of the same clinical relevance and importance for the patients. The multi-state approach proposed in this paper allows a separate investigation of treatment effects on the different components, and it seems to be important to compare both approaches with respect to interpretability of treatment effect estimation and power. Concerning power, the multi-state approach requires some kind of multiplicity adjustment as different treatment effects are estimated for the different components. Sequentially rejective test procedures provide a powerful and flexible tool to control type I error. As with other multivariate time to event outcomes, closed form solutions for sample size planning will be difficult to obtain [52], but simulation algorithms allow for an extensive investigation of sample size requirements, including for complex models [40, 52].

## Conclusion

This manuscript provides an accessible explanation of potential biases in treatment effect estimation when analysing composite endpoints. It illustrates that the risk for bias and its degree depend on whether first or multiple episodes per patient are analysed. Integrating multiple episodes into the statistical analysis model has the potential to reduce selection bias and to additionally capture indirect treatment effects. In particular for cardiovascular research, these findings may help to move beyond the paradigm of considering first events only.
